# Bycatch in Cetaceans from the North-Western Mediterranean Sea: Retrospective Study of Lesions and Utility of Bycatch Criteria

**DOI:** 10.3390/vetsci12080711

**Published:** 2025-07-29

**Authors:** Laura Martino, Mariona Leiva Forns, Marina Cid Cañete, Lola Pérez, Cèlia Pradas, Mariano Domingo

**Affiliations:** 1 Departament de Sanitat i Anatomia Animals, Facultat de Veterinària, Universitat Autònoma de Barcelona, 08193 Bellaterra, Spain; mariano.domingo@uab.cat; 2 Servei de Diagnòstic de Patologia Veterinària, Facultat de Veterinària, Universitat Autònoma de Barcelona, 08193 Bellaterra, Spain; mariona.leiva@autonoma.cat (M.L.F.); marina.cid@irta.cat (M.C.C.); 3 IRTA Programa de Sanitat, Centre de Recerca en Sanitat Animal (CReSA), Campus de la Universitat Autònoma de Barcelona, 08193 Bellaterra, Spain; 4 Unitat Mixta d’Investigació IRTA-UAB en Sanitat Animal, Centre de Recerca en Sanitat Animal (CReSA), Campus de la Universitat Autònoma de Barcelona, 08193 Bellaterra, Spain; 5 Facultat de Veterinària, Universitat Autònoma de Barcelona, 08193 Bellaterra, Spain

**Keywords:** necropsy, strandings, pathology, bycatch, fisheries, dolphin, cetacean, Mediterranean

## Abstract

Bycatch is the most common cause of death of small delphinids worldwide, including the Mediterranean Sea. The diagnosis of bycatch as cause of death in stranded cetaceans depends on the cumulative presence of multiple findings, termed bycatch criteria. In this study, we retrospectively evaluated the presence of bycatch criteria in 138 necropsied cetaceans, 136 stranded and 2 confirmed bycaught, in the Catalan Mediterranean Sea across a 13-year period. With the aim of identifying the most specific and reliable bycatch criteria, the animals’ cause of death was classified as either bycaught or other causes. Animals were necropsied according to standard procedures with complete histopathological examination and ancillary diagnostic techniques. We reviewed the necropsy reports and photographs of 138 cetaceans of seven species. Bycatch had been determined as the cause of death/stranding in 40 (29%) necropsied cetaceans. Both sexes were equally represented in the bycatch group. Bycatch was diagnosed in the Mediterranean common bottlenose dolphin (10/14; 71.4%), striped dolphin (29/108; 26.9%), and Risso’s dolphin (1/11; 9.1%). Sixty-seven out of 98 (68.3%) cetaceans that had been classified as non-bycatch had one or two bycatch criteria. Cetaceans with two and three major criteria had an overlap of causes of death, as some animals were diagnosed with bycatch and others with other causes of mortality. Animals with four criteria were invariably diagnosed as being bycaught. Recent feeding, absence of disease, good nutritional status, marks of fishing gear, multiorgan intravascular gas bubbles, hyphema and amputations or sharp incisions presumably inflicted by humans were significantly more likely to result in a diagnosis of bycatch, while loss of teeth and cranial fractures were not. None of the dolphins diagnosed as bycatch had ingested fishing gear. Our results highlight the relevance of bycatch as the cause of death of dolphins in the Mediterranean and suggest that some criteria traditionally linked to bycatch are not specific for bycatch in our region.

## Simple Summary

Bycatch (accidental capture in fisheries) is the most common cause of death of small delphinids worldwide. Determining bycatch to be the primary cause of death in a free-ranging stranded cetacean relies on the detection of lesions termed “bycatch criteria”, that vary in their specificity. Here, we retrospectively reviewed the bycatch criteria found in 138 necropsied cetaceans from the North-western Mediterranean Sea in a 13-year period to identify the most reliable criteria. Bycatch was determined as the cause of death/stranding in 40 (29%) of cetaceans. Both sexes were equally represented in the bycatch group. Bycatch was diagnosed in the Mediterranean common bottlenose dolphin (10/14; 71.4%), striped dolphin (29/108; 26.9%), and Risso’s dolphin (1/11; 9.1%). Recent feeding, absence of disease, good nutritional status, marks of fishing gear, intravascular gas bubbles, hyphema and amputations or sharp incisions presumably inflicted by humans were significantly more likely to result in a diagnosis of bycatch, while loss of teeth and cranial fractures were not. None of the dolphins diagnosed as bycatch had ingested fishing gear. Our results highlight the relevance of bycatch as cause of death of dolphins in the Mediterranean and show that some criteria traditionally linked to bycatch are not specific for bycatch in our region.

## 1. Introduction

Eleven cetacean species regularly inhabit the Mediterranean Sea, and some of them are under significant threats such as human interactions, disease, pollution, and climate change [1]. Fishery activities are the most significant threat affecting cetacean populations globally [2]. In a recent assessment for the Mediterranean and the Black Seas [3], five Mediterranean Sea resident cetacean species (*Balaenoptera physalus*, *Physeter macrocephalus*, *Globicephala melas*, *Delphinus delphis* and *Grampus griseus*) were assessed as endangered, one species (*Ziphius cavirostris*) as vulnerable, and a further species (*Steno bredanensis*) as near threatened. The other two resident species (*Tursiops truncatus* and *Stenella coeruleolba*) were rated as of least concern [4]. As expected for an area of high maritime traffic such as the Mediterranean Sea, risk for fin whales (*Balaenoptera physalus*) is posed mainly by ship strike and noise disturbance [5,6,7]. However, bycatch in fisheries is the main anthropogenic threat to smaller cetacean species worldwide [8]. Although the best assessment of bycatch is provided by on-board independent observers accounting for bycatch or by voluntary fishermen reporting and facilitating carcass necropsy [9], the continuous monitoring of strandings and investigation of the cause of stranding or death provide an additional estimation of threats to cetacean populations. In the Mediterranean Sea, there is evidence of bycatch affecting the common dolphin (*Delphinus delphis*), the common bottlenose dolphin (*Tursiops truncatus*), and the striped dolphin (*Stenella coeruleoalba*) [2,10,11]. Detection of bycatch at necropsy in stranded cetaceans is challenging, and the diagnosis is often based on the cumulative presence of various criteria and exclusion of other causes of death. Pathologists at the European Cetacean Society (ECS) discussed the criteria for diagnosis of bycatch in a workshop held in 1994 [12]. These criteria were later expanded to include evidence of decompression, with formation of gas bubbles, referred to as peracute underwater entrapment (PUE) [9,13,14,15]. Recently, a review of criteria for detection of bycatch yielded 25 criteria belonging to four basic groups [16]. Application of these criteria to bycaught harbor porpoises (*Phocoena phocoena*) in the North Sea [16] showed that some of these criteria may be nonspecific or not useful for this species or to this specific marine region. In the Catalan coast (north-western Mediterranean Sea), the causes of death and stranding of cetaceans are regularly monitored by necropsy, and bycatch is diagnosed by detection of commonly applied bycatch criteria, with minor modifications [10]. Herein, we review the cases diagnosed as bycatch on the Catalan coast during a period of 13 years (2012 to 2024), including two confirmed bycatch cases. We also analyze which criteria are more prevalent, specific, and potentially useful for bycatch diagnosis in our area.

## 2. Materials and Methods

### 2.1. Cetaceans Investigated and Necropsy Procedure

From January 2012 to December 2024, 138 cetaceans from 7 species (Table 1) were necropsied at the Veterinary Pathology Diagnostic Service (SDPV) of the Autonomous University of Barcelona (UAB) by ECVP certified pathologists, following established procedures [17,18]. All animals were stranded or bycaught in the Mediterranean coast of Catalunya, north-eastern Spain, along 580 km of coastline. Only cetaceans with a decomposition code of 1–3 (fresh to moderately decomposed) were subjected to postmortem study. A complete necropsy with histopathology was performed on each case, and samples were submitted for bacteriology or mycology when considered relevant. The body condition score of the carcass was determined by visual evaluation, as described elsewhere [19]. Samples for histopathology included lungs, lymph nodes, laryngeal tonsil, heart, longissimus dorsi, adrenal gland, spleen, liver, kidney, pancreas, stomachs, intestines, gonads, skin, encephalon, and spinal cord. Routine surveillance of cetacean morbillivirus (by immunohistochemistry and RT-PCR) and *Brucella* sp. (by PCR) were performed in all cases (see [10] for details on methodology). A computerized tomography (CT) scan was performed at the Veterinary Clinic Hospital of the UAB before necropsy, as a diagnostic aid, in 32 cetaceans during the period 2018–2024, whenever size of the animal and logistics allowed for it. Cases from 2012 to 2019 were included in a prior publication analyzing causes of cetacean mortality in our area [10].

Specific causes of death were introduced in a database and were classified in five general categories (bycatch, ship strike, infectious disease, non-infectious disease, and unknown). For the current study all cetaceans were retrospectively subjected to a deeper assessment of bycatch criteria detected at necropsy.

### 2.2. Criteria Used for Diagnosis of Bycatch

At necropsy, a protocol for diagnosis of bycatch was established based on the combination of proceedings of the ECS [12], the Spanish Cetacean Society [20] and more recent publications [15]. Following this combined protocol, bycatch diagnosis was established on the cumulative presence of at least three major criteria, either related to (i) the general health status of the stranded cetacean (good nutrition state, recent ingesta, absence of disease); (ii) detection of marks and lesions inflicted by fishing gear or presence of fishing gear itself; (iii) evidence of PUE as presence of gas bubbles in tissues and vessels (macroscopic and/or microscopic, and by CT); and (iv) presence of lesions, incisions, and amputations presumably inflicted by humans. Other minor bycatch criteria, classified as such because of their rareness among our necropsied cetaceans before 2012, were also considered, including fractures (cranium, maxilla and/or mandible) and segmental loss of multiple teeth [15,21], but alone they did not prompt the diagnosis of bycatch if at least three of the main criteria listed above were not also present. Other forms of human interaction, such as ship strike or propeller wounds, were detected macroscopically by wound evaluation. In rare cases, bycatch diagnosis was made when less than three criteria were present if the experience of the pathologists advised so [15,20].

The presence of gas bubbles was evaluated in a CT scan before necropsy, as well as grossly and histopathologically. The visualization of bubbles in multiple tissues, with any of these techniques, in the absence of putrefaction of the cadaver or post-mortem bacterial proliferation, was considered indicative of PUE.

**Figure 1:**
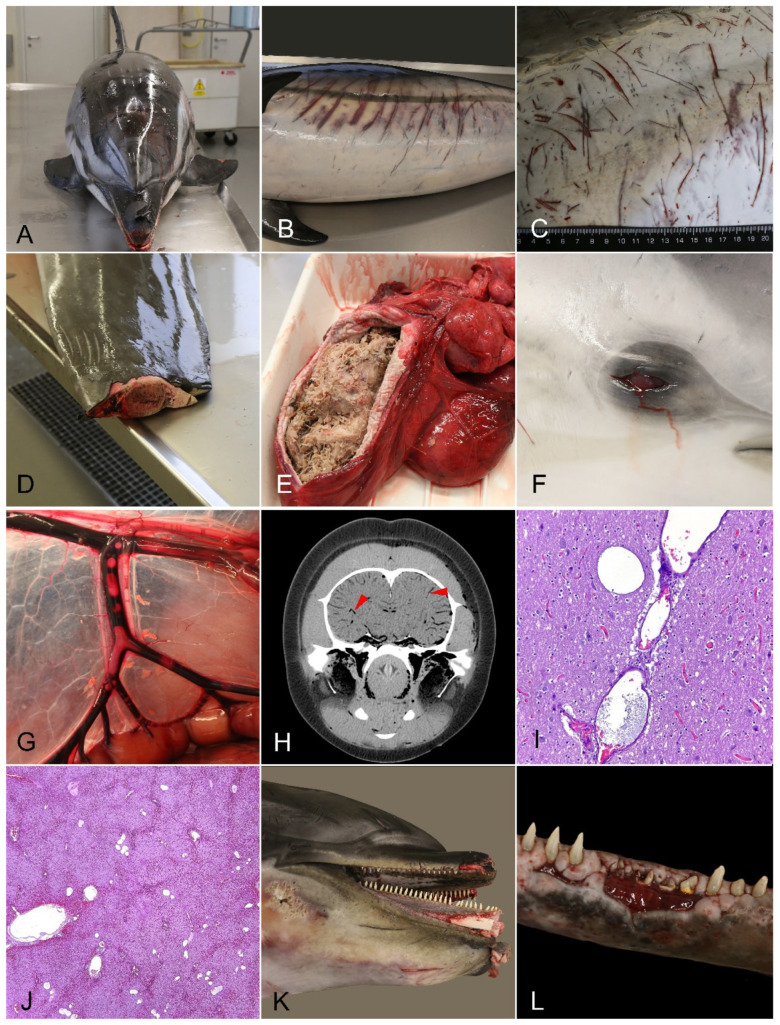
Criteria used to diagnose bycatch in the necropsied cetaceans in our protocol. (**A**) Good body condition. Case N-115/21. (**B**,**C**) Marks of fishing gear (scale bar = 1 cm). Cases N-8/24 and N-375/18, respectively. (**D**) Amputation. Case N-248/16. (**E**) Recent ingestion of prey in the forestomach. Case N-248/16. (**F**) Ocular redness (hyphema). Case N-42/18. (**G**–**J**) Intravascular gas bubbles at gross necropsy (**G**), CT scan ((**H**); red arrowheads) or histopathology ((**I**), brain; (**J**), liver). Case N-124/21 (**G**,**H**) and N-8/24 (**I**,**J**). (**K)** Fracture in the cranial or mandibular bones. Case N-414/24. (**L**) Selective loss of teeth. Case N-115/21.

For the retrospective analysis of the bycatch criteria applied on each case, the necropsy reports (*n* = 138), available photographs taken at necropsy, and CT scans were reviewed by two of the coauthors. Bycatch criteria were extracted from each necropsy report in cetaceans classified in all cause of death categories. Bycatch criteria detected with our protocol were tabulated categorically as present (“1”) or absent (“0”), and their frequency was analyzed in cetaceans diagnosed as bycatch or with other causes of death. Also, differences in the diagnosis of bycatch were analyzed by species, age and sex. The incidence of bycatch diagnosis was compared in two periods of time: 2012–2017, before implementation of a pre-necropsy CT scan, and 2018–2024, after its implementation. A definition and graphical summary of the studied bycatch criteria is shown in Figure 1 and Table 2. Some bycatch criteria suggested in the literature were not included, such as organ congestion, which has been more recently considered to be nonspecific [9]; pneumothorax, which is associated with severe pulmonary lesions in our cases [22]; and chyle in the mesenteric lymphatics which was replaced by the assessment of recent gastric content. Finally, the presence of pulmonary edema, formerly a bycatch criteria which is now considered to be nonspecific by some authors [8,16], was compared between animals diagnosed as bycatch and non-bycatch.

Sex, age group, species, and time period were compared in cetaceans diagnosed with bycatch and those with other causes of death using a 2-tailed P Fisher’s Exact test or a Chi-Square test (degrees of freedom = 1) with StatCalc tool, from EpiInfo (version 7.2.6.0). Significance was considered if *p* < 0.05. A Chi-Square test was used for comparing the frequency of pulmonary edema, recent ingestion, absence of disease, and good nutritional status, in bycatch and non-bycatch animals. Due to the small frequency of some criteria in the bycatch or non-bycatch group, a 2-tailed Fisher’s Exact test was used for ocular redness, loss of teeth, cranial fractures, amputations or sharp incisions, intravascular gas bubbles, presence of fishing gear or hooks, and marks of fishing gear.

## 3. Results

### 3.1. Causes of Death or Stranding

From the 138 cetaceans investigated, 54 (39.1%) were stranded alive and died shortly thereafter or were euthanized with sedation followed by an intravenous sodium pentobarbital overdose [25] following governmental permits. Human interaction was determined as the cause of death/stranding in 41 (29.7%) cetaceans, of which 40 (29% of total) were classified as bycatch with our protocol, and a single case died from ship strike. Two bycatch cases (N-90/20, a common bottlenose dolphin, and N-509/17, a striped dolphin) were delivered directly to port by fishermen. These two animals had, respectively, four and five major criteria. Both had a good nutritional status, recent ingestion of prey in the forestomach, and absence of other diseases. One had multiorgan intravascular gas bubbles, and the other marks of fishing gear and hyphema. Infectious and non-infectious diseases were diagnosed in 50 (36.2%) and 29 (21%) cases, respectively. The cause of death could not be established in 18 (13%) of the cetaceans (see Table 3). Complete information about each animal, with the final cause of death, biometric data and bycatch criteria observed, is available in Table S1.

### 3.2. Bycatch Criteria Detected

**Figure 2:**
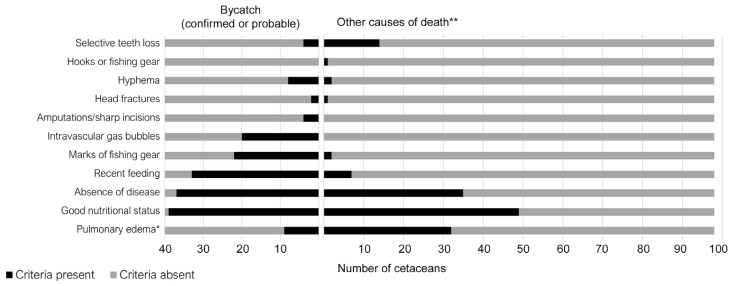
Presence or absence of bycatch criteria in necropsied cetaceans diagnosed as bycatch or other causes of death. Two dolphins were confirmed bycatch cases. * Traditional bycatch criteria, absent in our protocol, included only for comparison between groups. ** Other causes of death include animals classified as “unknown”.

Recent feeding, absence of evident disease, good nutritional status, marks of fishing gear, intravascular gas bubbles, hyphema and amputations or sharp incisions were significantly more prevalent in cetaceans diagnosed with bycatch as cause of death, with a *p*-value of <0.05 (see Table 4). However, half of the animals diagnosed with other causes of death had good nutritional status, and more than a third had no evident diseases. Finally, frequencies of cranial fracture, selective loss of teeth, and pulmonary edema were not significantly different between both groups. In fact, some of them were slightly more frequent in non-bycatch cetaceans (see Figure 2 and Table 4).

**Figure 3:**
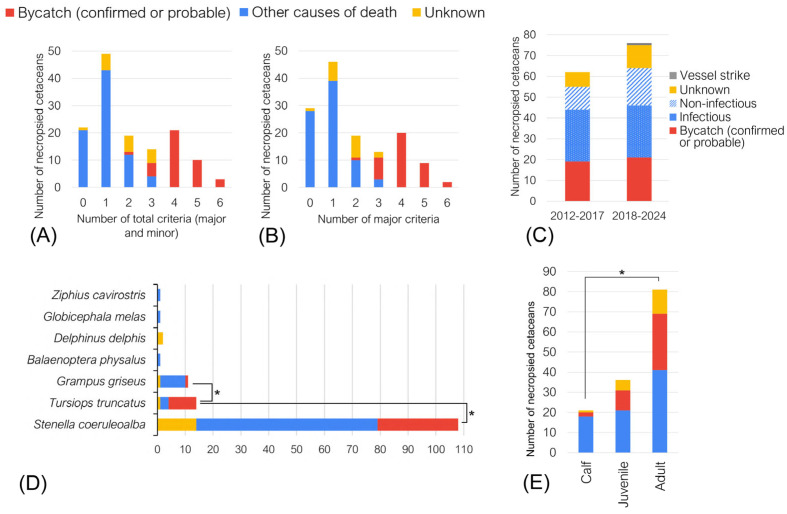
(**A**) Number of bycatch criteria detected in each cetacean distributed by the diagnosed cause of death. In all the figures, two confirmed bycaught dolphins are included in the bycatch group. (**B**) Number of major criteria distributed by cause of death. (**C**) Cause of death in the period before the implementation of pre-necropsy routine CT scan (2012–2017) and afterwards (2018–2024). (**D**) Distribution of bycatch diagnosis by species. * Statistical significance of bycatch compared to the other species (*p*-value < 0.005). (**E**) Distribution of strandings and bycatch diagnosis by age group. * Statistical significance (*p*-value < 0.05).

Cetaceans not diagnosed as bycatch (98/138) presented with between zero and three of the evaluated bycatch criteria in our protocol, both major and minor, although most (67/98) had only one or two criteria (Figure 3A). In most cases with bycatch diagnosis (34/40), there were four or more major and minor criteria present, and from those, thirteen had five or more criteria. Cetaceans with two or three bycatch criteria were diagnosed either as bycatch or as non-bycatch causes of death. In cetaceans with three criteria the diagnosis was bycatch (*n* = 5), mother–calf separation (*n* = 2), meningoencephalitis (*n* = 1), ship strike (*n* = 1) and unknown (*n* = 5).

Considering only the major criteria used in our protocol (Figure 3B), one dolphin with two major criteria was diagnosed as bycatch (a calf that had abundant milk in the stomach). In cetaceans with three major criteria (*n* = 13) causes of death were determined to be bycatch (*n* = 8), mother–calf separation (*n* = 2), unknown (*n* = 2), and ship strike (*n* = 1). Within the group of cetaceans with three bycatch criteria, absence of disease (7/8), recent feeding (7/8), marks of fishing gear (2/8), and gas bubbles (1/8), either alone or combined, were more frequent in cetaceans diagnosed as bycatch. In cetaceans classified with other causes of death, these criteria were found in 3/8, 2/8, 0/8 and 0/8 cetaceans, respectively (Table 5). The two cetaceans with three major criteria classified as “unknown” had absence of disease, recent feeding, and good nutritional status or hyphema. The eight dolphins with two major criteria classified as “unknown” had a high frequency of absence of disease and good nutritional status (7/8), and three of them had selective loss of teeth, considered a minor criterion.

Intravascular gas bubbles were detected in 9/32 CT scans performed (28.1%). All of these cetaceans were diagnosed as bycatch with ≥4 major criteria each. There were five dolphins without a previous CT scan diagnosed as bycatch by visualization of gas bubbles grossly at necropsy.

Intravascular multiorgan gas bubbles were observed histologically in 19/40 dolphins diagnosed as bycatch. In six dolphins that were also diagnosed as bycatch, gas bubbles were not observed grossly nor histologically.

### 3.3. Bycatch Diagnosis by Sex and Age Group

Of the 138 necropsied cetaceans, 67 were females (48.6%) and 71 males (51.4%). There was a similar proportion of both sexes in the group diagnosed as bycatch, with 21 females and 19 males. When considering striped dolphins, the predominant species, there were also no differences, with 14 males and 15 females classified as bycatch.

There were 81 adults (58.7%), 36 juveniles (26.1%), and 21 calves (15.2%). Adults were the age group with the most diagnoses of bycatch (*n* = 28; 70% of bycatch cases), followed by juveniles (*n* = 10; 25%), and calves (*n* = 2; 5%) (Figure 3E). The proportion of bycatch in adults was only significantly different when compared to calves (*p* = 0.031; Chi-square value 0.0248; degrees of freedom = 1), but not juveniles (*p* = 0.26; Chi-square value 0.52; degrees of freedom = 1).

### 3.4. Effect of CT Scan Use on Bycatch Diagnosis

From 2012 to 2017, bycatch was diagnosed in 30.6% of cetaceans, while after 2018, in 27.6% (Figure 3C). In one of the nine animals where intravascular gas in multiple organs was seen on CT scan, gas bubbles were not seen at necropsy.

### 3.5. Bycatch Diagnosis by Species

The species diagnosed as bycatch the most were striped dolphins, common bottlenose dolphins and Risso’s dolphins. The most frequently necropsied species was the Mediterranean striped dolphin, with 108 cetaceans, and the frequency of bycatch was high (29/108; 26.9%). The second in number was the common bottlenose dolphin, the one with the highest proportion of bycatch (10/14; 71.4%). This value was significantly higher (*p* < 0.005) when compared to striped and Risso’s dolphin (Figure 3D). The sample size of the other species was insufficient for comparison.

## 4. Discussion

In this study, we have retrospectively reviewed the diagnostic criteria met in the diagnosis of bycatch in necropsied stranded (*n* = 136) and bycaught (*n* = 2) cetaceans in Catalunya from 2012 to 2024, using our established necropsy protocol adapted to detect bycatch. We then analyzed the likeliness of each criterion for the diagnosis of bycatch. Our results show that the bycatch criteria that we classified as “minor”, such as loss of teeth and cranial fractures, did not increase the diagnosis of bycatch. On the other hand, a good nutritional status, recent ingested prey in the forestomach, lack of evidence of other diseases, marks of fishing gear, intravascular multiorgan gas bubbles, amputations or sharp incisions and ocular redness (hyphema) were significantly more frequent in establishing the diagnosis of bycatch.

Pulmonary edema, a criteria traditionally linked to accidental capture [15,21], was slightly more frequent in cetaceans not diagnosed as bycatch (32.7 vs. 22.5% among bycatch cases). Thus, as observed by others in different locations and species, pulmonary edema is not a specific criteria for bycatch and may reflect agony of different origins [8,16].

Selective loss of teeth, or broken teeth, was also not decisive when diagnosing bycatch. However, during the 13 years of case compilation, teeth were counted but not always described as broken or worn in the reports, so it is possible that some worn teeth have been misclassified as broken. In another study, in stranded cetaceans, there was a much higher proportion of broken teeth (43%) [23], while another study in bycaught short-beaked common dolphins the proportion was 63% [26].

Cranial fractures were rarely seen in our bycatch cases (5%), and differences were not significant in bycatch and non-bycatch groups. In North Sea harbor porpoises with confirmed bycatch, this was also a rare finding, with 1/11 porpoises having fractures [16]. However, other authors have found a 38% prevalence of mandibular fractures in strandings diagnosed as bycatch [23]. In bycaught dolphins in Australia, 43% of them had fractures [26]

In our cases, the body condition score was good in 97.5% of the bycatch dolphins, and in 50% of the non-bycatch cetaceans. A good body condition score was also a common finding in stranded delphinids from the Canary Islands diagnosed as bycatch [23]. In a study from the US 13/14 confirmed bycaught cetaceans, including short-beaked common dolphins, harbor porpoises, and Atlantic white-sided dolphins (*Lagenorhynchus acutus*), had a robust body condition [9], and this was also the case in bycaught dolphins in Australia [26]. These results differ from those of confirmed bycaught harbor porpoises, that had varied body condition scores, ranging from emaciated to normal, and more than half had previous debilitating diseases [16]. These contradictory studies raise the question of whether a good body condition should be invariably counted as a bycatch criterion. The lack of pathological studies in confirmed bycaught striped dolphins, the predominant species in our coastline, leaves this question open.

Recently ingested prey in the forestomach of dolphins diagnosed as bycatch was significantly higher than in cetaceans diagnosed with other causes of death (82.5 vs. 7.1%). This is consistent with findings in the Northeast coast of the US [9], the Canary Islands [23], South Australia [26], and the North Sea [16] in different cetacean species. It is true, however, that bycaught cetaceans can potentially have empty stomachs because of regurgitation or digestion of ingested prey, and the presence of stomach contents is regarded as an indicator of acute death [9,23]. In our dataset, there was one dolphin calf diagnosed as bycatch by two bycatch criteria. As milk is not prey, it was not counted as “recent feeding” in our retrospective analysis. In the future, milk ingestion may be included as a bycatch criteria, as it indicates sudden death, and calves are reported to suffer from bycatch in short-beaked common dolphins and harbor porpoises [9,26].

Cutaneous marks compatible with fishing gear interaction were seen in 55% of our dolphins diagnosed of bycatch, and only in 2% of the non-bycatch group. Cutaneous marks in the non-bycatch group are of uncertain origin and presumably occurred post-mortem or pre-mortem by scratching against sand or rocks. Our proportion of animals with cutaneous marks is slightly lower than in other publications with confirmed bycatch cetaceans; in the Northeast coast of the US it was about 70% [9], and in Australia, 82% of short-beaked common dolphins had net marks [26]. Similarly, in the North Sea, all bycaught porpoises had net marks [16]. The authors speculate that fishing techniques influence the direct interaction of the bycaught cetaceans and therefore that in the Mediterranean Sea these lesions are not that frequent. Other possibilities are that our sample size is too small or that some marks fade over time.

Ocular redness (hyphema) was more frequent in dolphins diagnosed with bycatch (20 vs. 3.1%). Other authors have found significant differences between bycaught and non-bycaught cetaceans, but with a higher proportion in bycaught cetaceans (about half of them) [9,16]. In contrast, another study has found this lesion to be rare in stranded dolphins diagnosed with bycatch [23]. Overall, although not very frequent, hyphema could be a useful criterion of bycatch in our area.

Intravascular gas bubbles in multiple tissues were found in half of the cases diagnosed as bycatch and in none of those diagnosed with other causes of death. Significant differences were also found in a study conducted in the Northeastern US coast with confirmed bycatch cetaceans [9], and it was also frequent in another publication with stranded cetaceans diagnosed with bycatch [23]. Conversely, in a study in the North Sea, there was only 1/11 porpoises with gas bubbles in a lymph node [16], and gas bubbles were absent in bycaught short-beaked common dolphins in Australia [26]. This probably indicates differences in the bycatch-associated lesions depending on the species of cetacean, type of fishing gear and depth.

Pneumothorax has been mentioned as an indicator of bycatch [27]. Dolphins with tension pneumothorax in our dataset (*n* = 4), however, had zero or one bycatch criteria and there were severe pulmonary lesions in three of them [22], making it an unlikely indicator of bycatch by itself, as indicated by others [16].

In our study, most of the animals diagnosed as bycatch presented with at least four criteria, considering both major and minor criteria used in our protocol. Cetaceans with two and three major or minor criteria were the most problematic group, as some of them were diagnosed as bycatch while others were not, including twelve cetaceans with unknown cause of death. Absence of disease, recent feeding, marks of fishing gear and intravascular gas bubbles were determinant in the three-criteria group to classify an animal as bycatch. Since there were animals with three bycatch criteria deemed non-bycatch deaths, we recommend, in our necropsy protocol for our area, reducing the level of certainty for cases with three major criteria to “suspected”, especially if the alternative is diagnosed as unknown cause of death, while those with four criteria or more are classified as “probable” cases.

Our assessment of bycatch, in general terms, is similar to more recent guidelines elaborated by Pietroluongo et al. [28]. We did not base our protocol for bycatch diagnosis on these guidelines because they were published in 2021 and our necropsies started in 2012. Therefore, our protocol did not consider recently described bycatch criteria included in the guidelines, such as separation of rectus abdominis muscle [29]. Some discordances were found. For example, the presence of fractures, considered a “consistent” finding in that guideline, was considered a minor criterion and did not prompt a diagnosis of bycatch in the absence of other primary criteria. Also, the guideline does not establish a threshold of “consistent” criteria for a “probable” diagnosis of bycatch. Moreover, the scientific evidence on which Pietroluongo et al. was based includes a low representation of striped dolphins. As discussed above, according to publications with confirmed bycatch cetaceans, there are notable variations in the frequency of bycatch criteria such as marks of fishing gear, body condition score, fractures, loss of teeth, hyphema and gas bubbles in different species, location and fishery techniques. In the future, more research must focus on these specific variations to hopefully provide standardized guidelines for each region.

Most of the anthropogenic interactions diagnosed in this publication as a cause of death (40/41) were consistent with bycatch. No dolphins in this dataset were documented as entangled with or having ingested fishing gear or with evidence of fisherman aggression (stab wounds, gunshots, etc.) as has been documented in other areas [23]. There was a single cetacean that died from erysipelas that had an incidental hook ingestion [30], and only one case of ship strike in a Cuvier’s beaked whale [10].

Among the studied species, common bottlenose dolphins *Tursiops truncatus* had the highest proportion of bycatch as a cause of death (71.4%). This is a recognized threat to this species in the Mediterranean, although the conservation status is of least concern [4]. *S. coeruleoalba* were the most numerous species in this dataset and had more varied causes of death than other species, a significant proportion of which were due to bycatch (26.9%). The higher absolute numbers are probably derived from the abundance of the species in the Mediterranean [4].

Risso’s dolphins were underrepresented in our dataset, and only one animal (1/11; 9.1%) died from bycatch. Although the low sample size precludes taking conclusions, fishery interaction does not seem the primary threat to this species in our area, as the main cause of death recorded in our necropsies is sinusitis by *Crassicauda grampicola*. Decompression sickness, however, has been described in the Canary Islands attributed to a rapid change in water depth during the hunting of a squid by a Risso’s dolphin [31]. More necropsies need to be performed in Risso’s dolphins in our area to detect potential anthropogenic threats to this endangered species.

Short-beaked common dolphins (*Delphinus delphis*) are of particular concern in the Mediterranean region due to their dramatic population decline in recent decades [4,32]. The only two animals necropsied in this period in our facilities had unknown cause of death, mainly due to a lack of enough bycatch criteria, with a total of three criteria each, two major and one minor. They had good body condition, no evidence of disease and selective loss of teeth. Both had ingested strange material (one plastic, the other plant material). These findings are similar to those reported in Australia [26], where the most common gross findings were broken teeth, net marks, and a good body condition, in animals confirmed to by bycaught. Further studies need to be performed in short-beaked common dolphins of the Mediterranean to detect potential differences in the expression of bycatch criteria, either due to differences in diving behavior and/or type of fishing gear involved, to avoid missing evidence of fishery interaction.

Large cetaceans were underrepresented in our case series. However, fin whales are known to be impacted by vessel collision, both in Italy and Catalunya [5,6]. Smaller cetaceans are not as frequently reported being struck by vessels, and our results are also consistent with this.

The age and sex of the animals did not differ in bycatch vs. non-bycatch group. In the Canary Islands, no significant differences between age groups were found in dolphins [23]. In the North-eastern Atlantic, bycaught common and striped dolphins tended to be male and juveniles [33]. In the western Mediterranean, drifting nets caught more striped dolphin males [11]. Other authors have found that juveniles and females of *Delphinus delphis* are the most affected group [26]. These differences may be attributable to behavioral patterns and fishing practices, or to the fact that some of these studies were performed in confirmed bycaught cases and not in stranded animals, which makes comparison difficult.

In our diagnostic service, the implementation of a CT scan prior to necropsy in fresh carcasses did not increase the diagnosis of bycatch, as the total count of criteria was enough to make the diagnosis even without evidence of gas embolism. However, in our experience, CT scan is the most reliable tool to detect intravascular gas bubbles in a fresh carcass and strongly supports evidence of PUE that helps in the diagnosis of bycatch. Moreover, CT scan may increase the sensitivity for gas detection, as in one of the cases where gas was visualized in the scan, bubbles were not seen at necropsy.

Only 2 of 138 cetaceans in this study were confirmed to be bycaught by fishermen who brought the dolphins to port. The other 38 cetaceans diagnosed as bycatch were inferred based on necropsy findings without previous signalment. To this date, there are no publications that define bycatch criteria in the striped dolphin or common bottlenose dolphin using confirmed bycatch cases, so specific or more prevalent lesions of bycatch in these species are missing. Additionally, our results may not be representative of bycatch criteria in short-beaked common dolphins of the Mediterranean, since we only necropsied two in this period. Ideally, future collaborations between the stranding network, veterinary pathologists and fishermen reporting bycaught animals and facilitating carcass necropsies will help establish reliable and specific lesions of bycatch in our area with the predominant delphinid species.

## Figures and Tables

**Table 1:** Necropsies performed distributed by year and species.

Species/ Year	2012	2013	2014	2015	2016	2017	2018	2019	2020	2021	2022	2023	2024	Total
*B. physalus *				1										1
*D. delphis *						1				1				2
*G. melas *										1				1
*G. griseus *	1	2			5			1		1			1	11
*S. coeruleoalba *	14	1	6	6	5	16 ^1^	18	5	8	14	2	6	7	107
*T. truncatus *	3				1		1		2 ^1^	2	2	2	1	14
*Z. cavirostris *								1 ^2^						1
Total	18	3	6	7	11	17	19	7	10	19	4	8	9	138

^1^ Includes one confirmed bycatch case brought to port by fishermen (total *n* = 2). ^2^ Ship strike.

**Table 2:** Bycatch diagnosis protocol used in necropsies reviewed in this publication with definition of the retrospectively studied bycatch criteria in the 138 cetaceans. The right column indicates the corresponding picture in Figure 1.

Diagnostic Category	Bycatch Criteria	Definition	Figure 1
Major criteria: Probable bycatch diagnosed if ≥3 *	**Good nutritional status**	Normal body condition score, with round or convex epiaxial area [19]; normal blubber thickness and subcutaneous adipose tissue	A
	**Marks of fishing gear**	Cutaneous marks suggestive of interaction with fishing ropes or nets [15,18,23]	B, C
	**Amputations/sharp incisions**	Amputation of the fluke, fins, or other parts of the body; sharp incisions or perforations [15]	D
	**Recent feeding**	Undigested or partially digested prey in the forestomach [12]	E
	**Hyphema**	Reddened eye, with or without eye bulging (proptosis), due to hemorrhage in the anterior chamber [9]	F
	**Evidence of PUE (intravascular gas bubbles)**	Multiorgan intravascular gas bubbles in the CT scan, gross necropsy or histopathology, in fresh carcasses without evidence of putrefaction [14,24]	G, H, I, J
	**Hooks or fishing gear**	Entangled/ingested fishing gear or presence of hooks	ns
	**Absence of disease**	No evidence of other diseases that explain stranding or death	ns
Minor criteria: support diagnosis of bycatch, but only if ≥3 of the above are present	**Head fractures**	Fractures in the cranial or mandibular bones [12]	K
	**Selective teeth loss**	Loss of consecutive teeth by sharp fracture [21], not attributed to age wear	L

* Bycatch could be diagnosed with less than three major criteria if the experience of the pathologist advised so. ns = not shown in Figure 1; see Table S1.

**Table 3:** Frequency of causes of death or stranding in cetaceans necropsied in the study period, classified in general etiologic groups (detailed data available in Table S1).

Cause of Death or Stranding	n	%
Bycatch *	40	29
Vessel strike	1	0.7
Infectious disease	50	36.2
Non-infectious disease	29	21
Unknown	18	13
Total	138	100%

* Includes two confirmed bycatch dolphins.

**Table 4:** Frequency of detection of bycatch criteria in necropsied cetaceans with the bycatch protocol for this analysis. Bold: significantly higher frequency in cetaceans diagnosed as bycatch. * Traditional bycatch criteria, absent in our protocol, included only for comparison between groups. ** Other causes of death include animals classified as “unknown”. Degrees of freedom for the Chi-square tests were 1 in all criteria.

		Diagnosis of Bycatch		Other Causes of Death **		Significance	Chi-Square
	Criteria for Detection of Bycatch	n	%	n	%	*p*	Value
Major criteria	Good nutritional status	39/40	97.5	49/98	50	**<0.0001**	27.74
	Absence of disease	37/40	92.5	35/98	35.7	**<0.0001**	36.71
	Recent feeding	33/40	82.5	7/98	7.1	**<0.0001**	77.64
	Marks of fishing gear	22/40	55	2/98	2	**<0.0001**	55.43
	Intravascular gas bubbles	20/40	50	0/98	0	**<0.0001**	57.31
	Amputations/sharp incisions	4/40	10	0/98	0	**<0.05**	10.09
	Hooks or fishing gear	0/40	0	1/98	1	0.522	0.41
	Hyphema	8/40	20	3/98	3.1	**<0.002**	11.11
Minor criteria	Head fractures	2/40	5	1/98	1	0.201	2.11
	Selective loss of teeth	4/40	10	14/98	14.3	0.586	0.52
Other	Pulmonary edema *	9/40	22.5	32/98	32.7	0.222	1.49

**Table 5:** Comparison of major and minor bycatch criteria detected in cetaceans with two and three major criteria, in absolute numbers. Bc = bycatch. Other = other causes of death. U = unknown.

		2 Criteria			3 Criteria		
	Criteria	Bc	Other	U	Bc	Other	U
Major criteria	Good nutrition	1/1	10/10	7/8	7/8	3/3	1/2
	Absence of disease	1/1	7/10	7/8	7/8	3/8	2/2
	Recent feeding	0/1	1/10	0/8	7/8	2/8	2/2
	Marks of fishing gear	0/1	1/10	0/8	2/8	0/8	0/2
	Intravascular gas bubbles	0/1	0/10	0/8	1/8	0/8	0/2
	Amputations/sharp incisions	0/1	0/10	0/8	0/8	0/8	0/2
	Hooks or fishing gear	0/1	0/10	0/8	0/8	0/8	0/2
	Hyphema	0/1	1/10	0/8	0/8	1/8	1/2
Minor criteria	Head fractures	0/1	0/10	0/8	0/8	0/8	0/2
	Selective loss of teeth	0/1	1/10	3/8	3/8	0/8	0/2

## Data Availability

See Table S1 for tabulated bycatch criteria and additional information about all cetaceans included in this study.

## Supplementary Materials

The following supporting information can be downloaded at https://www.mdpi.com/article/10.3390/vetsci12080711/s1, Table S1: Stranded cetaceans.

## Abbreviations

The following abbreviations are used in this manuscript:

CT	Computered Tomography
